# Epicardial cell activation as a paradigm shift in cardiac repair and regeneration

**DOI:** 10.1093/stcltm/szaf074

**Published:** 2026-01-30

**Authors:** Roberto Vanni, Matteo Aubry, Mauro Rinaldi, Raffaella Rastaldo, Claudia Giachino

**Affiliations:** Department of Clinical and Biological Sciences, University of Turin, Turin 10043, Italy; Department of Clinical and Biological Sciences, University of Turin, Turin 10043, Italy; Department of Surgical Sciences, University of Turin, Turin 10126, Italy; Department of Clinical and Biological Sciences, University of Turin, Turin 10043, Italy; Department of Clinical and Biological Sciences, University of Turin, Turin 10043, Italy

**Keywords:** cardiac repair, epicardium, epithelial-to-mesenchymal transition, innovative therapies, myocardial infarction

## Abstract

Cardiovascular diseases, particularly myocardial infarction, remain a leading cause of mortality globally, primarily due to the adult heart’s limited regenerative capacity. Recent discoveries have highlighted the epicardium, a mesothelial layer surrounding the heart, as a critical player in cardiac repair and regeneration. During development, the epicardium plays a central role in heart formation by providing progenitor cells, structural components, and paracrine signals. Emerging evidence indicates that this developmental potential can be reactivated in the adult heart following injury. Upon activation, epicardial cells undergo epithelial-to-mesenchymal transition, proliferate, and secrete a range of paracrine factors that influence angiogenesis, inflammation resolution, and extracellular matrix remodeling. This review explores the mechanisms underlying epicardial activation, its contributions to heart development and myocardial repair, and its therapeutic potential. We discuss small molecule modulators, gene therapy, cellular therapies, and biomaterial-based approaches that aim to harness the regenerative capacity of the epicardium. These approaches, which move beyond scar tissue formation to possible regeneration, have the potential to transform the landscape of cardiac regenerative medicine. Despite promising preclinical results, however, challenges such as interindividual variability, incomplete differentiation of epicardial-derived cells, and delivery constraints must be addressed. Advances in single-cell technologies, biomaterial engineering, and translational research are paving the way for personalized and effective epicardium-based therapies. By redefining the role of the epicardium in cardiac biology, epicardial activation offers a novel paradigm for treating ischemic heart disease and heart failure.

## Graphical abstract

**Graphical Abstract:**
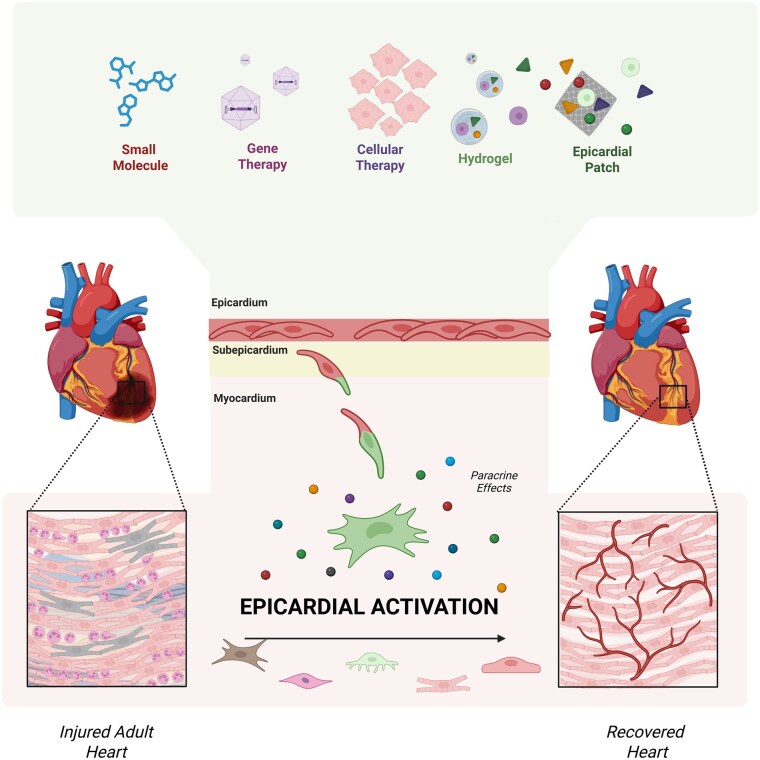

Significance statement Recent discoveries have highlighted the epicardium, a mesothelial layer surrounding the heart, as a critical player in cardiac repair and regeneration. During development, the epicardium plays a central role in heart formation by providing progenitor cells, structural components, and paracrine signals. Emerging evidence indicates that this developmental potential can be reactivated in the adult heart following injury. This concise review synthesizes current knowledge on the mechanisms and therapeutic applications of epicardial activation, emphasizing its transformative potential in cardiac regenerative medicine.

## Introduction

In recent years, the epicardium, a mesothelial layer enveloping the heart, has emerged as a promising therapeutic target for cardiac repair and regeneration.^1^ Once thought to be quiescent in adults, the epicardium has demonstrated the ability to reactivate following cardiac injury, mimicking its dynamic role during embryonic heart development.

Epicardial activation is thus emerging as a paradigm shift in regenerative medicine, moving beyond conventional approaches focused solely on cardiomyocytes (CMs). It highlights the importance of all the heart’s structures in orchestrating complex repair processes. This perspective aligns with the broader concept of the heart as an integrated organ system, where non-myocyte components of epicardial origin play critical roles in maintaining cardiac homeostasis and facilitating recovery after injury.^2^

This concise review synthesizes current knowledge on the mechanisms and therapeutic applications of epicardial activation, emphasizing its transformative potential in regenerative medicine. By moving beyond scar stabilization to true myocardial regeneration, epicardium-based therapies represent a paradigm shift in the treatment of ischemic heart disease and heart failure. Future directions in epicardial research, including single-cell transcriptomics, advanced biomaterials, and integrative regenerative strategies, hold promise for overcoming existing barriers and translating these findings into clinical success.

## The epicardium and its role in heart development

During embryogenesis, the epicardium arises from the proepicardial organ and contributes to heart formation by providing signaling molecules, progenitor cells, and structural support.^3^ Located near the venous pole of the embryonic heart, the proepicardial organ generates cells that migrate to envelop the myocardium, forming the epicardium. In addition to serving as a protective layer, the epicardium is instrumental in cardiac development. During development, a specific subset of epicardial cells undergoes a transformation known as epithelial-to-mesenchymal transition (EMT). This process results in the formation of epicardial-derived cells (EPDCs), which then migrate into the myocardium. EPDCs predominantly differentiate into fibroblasts and mural cells in the heart, including vascular smooth muscle cells and pericytes.^3^ The debate continues regarding whether EPDCs give rise to CMs and coronary endothelial cells, as studies present conflicting evidence.^4^^,^^5^ It is, however, possible that some epicardial cells may enhance cardiogenic-like gene programs, as has been reported for cardiac fibroblasts.^6^ Also in consideration of the fact that resident cardiac fibroblasts often originate from EPDCs.^7^ Beyond direct differentiation, epicardial cells secrete paracrine factors that influence cardiac development and repair. These factors promote CM proliferation, modulate extracellular matrix (ECM) composition, and facilitate neovascularization, thereby supporting myocardial growth.^8^^,^^9^ EPDCs are thus essential for the structural and functional maturation of the heart, initially functioning as a simple epithelial sheet and later undergoing a transition to a more dynamic structure capable of influencing adjacent tissues.^3^ Recently, single-cell transcriptomics and chromatin accessibility profiling analyses of human epicardioids have provided insights into the molecular mechanisms guiding this process, revealing key transcriptional networks involved in epicardial cell differentiation and function.^10^ Disruptions in epicardial signaling pathways can lead to congenital heart defects (CHD),^11^^,^^12^ evidencing their critical roles. Several animal models, displaying anomalies in the formation of the epicardium or EPDCs, show phenotypes that can be linked to the pathophysiology of different types of CHD. Examples include Holt–Oram syndrome, an autosomal dominant condition caused by mutations in the TBX5 gene and characterized by structural cardiac abnormalities, the commonest including atrial and/or ventricular septal defects.^13^ This syndrome could be caused by anomalies in the main roles normally played by EPDCs, their contribution to coronary development, and the production of paracrine growth factors for the myocardium. Another example is Meacham syndrome, characterized by complex congenital heart disease and diaphragmatic abnormalities. The only genetic anomaly hitherto associated with this syndrome has been mutations in the Wilms’ tumor suppressor gene (WT1).^14^ Since Wt1 is expressed in the embryonic epicardium, the authors of this study suggested that the cardiac defects observed in patients with Meacham syndrome, mainly ventricular and atrial septal defects, could be related to the defective development of the epicardium and EPDCs. Further, a few animal models of defective epicardial development show a thinning of the ventricular wall, a phenotype that might be related to the human condition known as left ventricular non-compaction. Moreover, β-catenin deletion in the proepicardium results in a thin ventricular myocardium and embryonic death^15^ while loss of function of the mucin-like transmembrane glycoprotein podoplanin, expressed by the epicardium, causes defective epicardial development together with a hypoplastic, perforated, compact septal myocardium.^16^ The epicardium has also been considered relevant to the pathophysiology of arrhythmogenic right ventricular cardiomyopathy, a congenital disorder that causes progressive replacement of the right ventricular myocardium by fibrofatty tissue.^17^

Of note, the epicardium’s role in cardiac development is conserved across all vertebrates, underscoring its evolutionary significance. These developmental and evolutionary insights form the foundation for understanding the epicardium’s regenerative potential in adults.

## Epicardial activation following cardiac injury

Similar to heart development, in response to cardiac injury in the adult heart, the activated epicardium undergoes EMT to produce EPDCs that participate in tissue repair.^18^^,^^19^

Numerous investigations highlighted that epicardial reactivation is an evolutionarily preserved process. However, its efficiency varies widely across species, ranging from regeneration of adult heart portions in lower vertebrates to a narrow reparative window limited to the early postnatal days in small mammals.^20^

### Mechanisms of epicardial activation in mammals

Following cardiac damage, such as MI, the adult epicardium can reactivate genes expressed during embryonic development, upregulating transcription factors like WT1 and *T* box 18 (Tbx18).^21^^,^^22^ This activation is initiated by injury-induced signaling pathways that stimulate epicardial cell proliferation, EMT, and paracrine factor secretion.^3^ Epicardial EMT allows cells to acquire mesenchymal characteristics, enhancing their motility and differentiation potential. This process is regulated by injury-induced signals such as transforming growth factor-beta (TGF-β), Wnt/β-catenin, and Hedgehog (Hh) pathways.^23^ Activated epicardial cells proliferate and migrate into the myocardium, where they participate in tissue remodeling and repair. This response is essential for replacing damaged cells and restoring cardiac function.^24^ Interestingly, recent studies in the zebrafish^25^ and infarcted mouse hearts^26^ suggest that an EMT-like response may occur not only in epicardial cells but also in CMs,^25^ confirming the critical role of EMT-like mechanisms in cardiac regenerative processes. Beyond cell-autonomous roles, epicardial cells release a range of growth factors and cytokines that influence adjacent cell types. The epicardial paracrine release is further reinforced by recent studies utilizing transcriptomic technologies.^27^^,^^28^ These paracrine effects are vital for creating a reparative environment following myocardial infarction.^3^ For instance, vascular endothelial growth factor (VEGF) and fibroblast growth factor (FGF) improve angiogenesis, while interleukin-6 (IL-6) modulates inflammatory responses.^29^ A dual effect of IL-6 has been observed depending on the timing of IL-6 signaling activation. In the acute phase post-MI, IL-6 can induce CM dedifferentiation, thus initiating cell proliferation and contributing to functional recovery.^30^ Early work had already underscored that even partial inactivation of gp130, a critical component of the IL-6 signaling, profoundly suppressed CM proliferation and survival.^31^ IL-6 signaling also reduces CM apoptosis and promotes survival, which is essential for early tissue preservation.^32^^,^^33^ However, prolonged IL-6 signaling well beyond the initial injury worsens cardiac function by reducing contractility and activating the hypertrophic gene program.^32^

**Figure 1.**
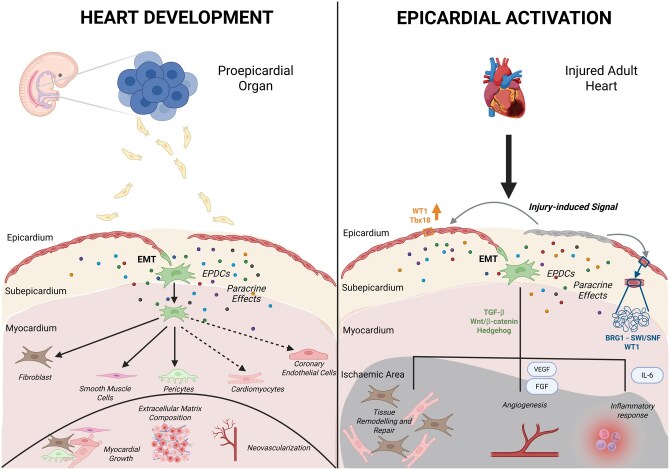
Epicardial plasticity. Role of the epicardium during heart development (left) and in response to myocardial infarction (right). During embryogenesis, cells from the proepicardial organ migrate to envelop the myocardium, forming the epicardium. A subset of epicardial cells undergoes epithelial-to-mesenchymal transition (EMT), generating epicardial-derived cells (EPDCs) that invade the myocardium and differentiate primarily into fibroblasts, vascular smooth muscle cells, and pericytes. Their potential to form cardiomyocytes and coronary endothelial cells remains a matter of ongoing debate (dotted line). Beyond direct differentiation, the epicardium contributes to heart development by secreting paracrine factors that promote cardiomyocyte proliferation, regulate extracellular matrix remodeling, and support coronary vessel formation. In the adult heart, following myocardial infarction, injury-induced signals reactivate the adult epicardium, upregulating developmental transcription factors such as WT1 and Tbx18, along with epigenetic regulation via the BRG1–SWI/SNF complex, triggering EMT, epicardial proliferation, and the release of paracrine factors. Key signaling pathways govern EPDC activation and the release of VEGF, FGF, and IL-6. These signals promote angiogenesis, inflammation modulation, and cell proliferation and differentiation in the ischemic myocardium, reflecting an evolutionarily conserved mechanism of cardiac repair. Created in https://BioRender.com.

Epigenetic mechanisms also play a role in epicardial activation. The BRG1–SWI/SNF chromatin remodeling complex regulates the Wt1 locus, influencing epicardial cell behavior during heart development and repair.^34^ SMARCA4 (SWI/SNF-related, matrix-associated, actin-dependent regulator of chromatin, subfamily a, member 4), the core catalytic subunit of the chromatin remodeling complex, has the potential to target some reactivated epicardial genes in MI. Interestingly, epicardial SMARCA4 deletion in adult mice was found to exacerbate cardiac injury following MI, due to the inhibition of EMT and differentiation of EPDCs.^35^ To further understand epicardial activation, innovative models such as epicardial slices and heart-on-a-chip have been developed.^36^^,^^37^ These 3D organotypic models replicate the multicellular complexity of the epicardial environment, providing valuable insights into its role in cardiac repair. Human epicardial cells can express epicardial markers and undergo EMT similarly to other species. However, unlike murine models, which exhibit a monolayered epicardial epithelium, the human epicardium during development is characterized by a multilayered structure.^34^ Further investigation is needed to uncover the interspecies differences in epicardial activation (Figure 1).

### Signaling pathways regulating epicardial–myocardial communication in mammals

Once activated, the epicardium secretes multiple signaling molecules regulating the communication with the myocardium, thereby promoting heart repair and regeneration.^1^^,^^9^

Retinoic acid (RA) signaling plays a crucial role in the function of the epicardium. Disruption of RA signaling can result in defects in myocardial growth and coronary vessel formation.^38^

Indeed, the involvement of RA receptors like RXRα was essential for these processes in the murine epicardium.^39^ RA signaling reactivates epicardial progenitors, promoting their differentiation into vascular smooth muscle cells and enhancing vascular regeneration.^40^ During mouse heart development, the expression of retinaldehyde dehydrogenase 2 (Raldh2), the rate-limiting enzyme for RA production, was present in the epicardial layer, while it was absent in the adult ventricular wall. Interestingly, this embryonic gene was re-induced transiently (2 weeks after MI) in adult mouse epicardium, even if at a lower level, with concomitant appearance of mesenchymal cells in the sub-epicardium expressing some embryonic genes.^41^ The potential role of RA signaling in mammalian cardiac regeneration remains to be thoroughly examined.

FGFs, particularly FGF1, FGF2, FGF9, and FGF16, are expressed in the epicardium and play crucial roles in cardiac development. They promote cell survival, proliferation, and differentiation by acting downstream of RA signaling.^38^ Co-culture experiments revealed that epicardial cells secrete FGFs that stimulate the proliferation of myocardial cells. In an *in vivo* rat model, MI increased FGF1 levels in inflammatory cells and fibroblast-like cells located within the border zone of the infarcted area.^42^ Conversely, FGF2 expression was upregulated in endothelial cells within the border zone and in CMs located in the infarcted myocardium.^42^ Since FGF receptor signaling is essential for the normal response to MI in rat hearts, the therapeutic applications of FGF in cardiovascular disease have been widely explored.^43^ Nevertheless, the involvement of this pathway in the epicardial activation of cardiac injury in mammals deserves further studies.

Signaling through both platelet-derived growth factor **(**PDGF) receptors is crucial for the process of epicardial EMT,^44^ possibly via NF-κB activation.^45^ After MI, *Pdgfb*, *Pdgfra*, and *Pdgfrb* were upregulated in the infarcted region of mouse hearts, and the phosphorylation of PDGFRβ in perivascular cells within the infarct zone was an indicator of active signaling.^46^ Antibody-mediated PDGFRα and PDGFRβ inhibition attenuated collagen deposition and vessel maturation in the infarcted area,^46^ further demonstrating their healing role.

TGFβ signaling also plays a crucial role in inducing the EMT by downregulating epithelial markers and upregulating mesenchymal markers,^47^ thereby contributing to cardiac repair.^11^^,^^48^ Activation of TGFβ pathways promotes the migration and differentiation of epicardial cells into various heart cell types.^49^ This process has been identified as a primary mediator of oxytocin-induced epicardial activation.^50^ Infarcted adult rat and swine hearts exhibit a marked induction of *Tgfb1*, *Tgfb2*, and *Tgfb3.*^51-53^ Considering the multiple roles of TGFβ signaling in cardiovascular development and disease,^54^ this pathway should be systematically studied to elucidate cell-type-specific and isoform-specific functions following cardiac injury.

Similarly, the Wnt/β-catenin signaling plays a relevant role in epicardial activation and EMT. Activation of this pathway in the epicardium leads to increased proliferation and differentiation of epicardial cells, ultimately contributing to myocardial repair and regeneration.^15^^,^^49^ Inhibiting Wnt signaling has been shown to impair epicardial activation and repair.^15^ In a mouse model, the induction of *Wnt1* expression was observed in both the epicardium and cardiac fibroblasts following infarction.^55^ β-catenin deficiency in epicardial cells hindered their growth and EMT, as well as impaired cardiac performance post-MI. Additionally, β-catenin deficiency in adult cardiac fibroblasts resulted in acute cardiac dilatation and dysfunction.^55^ In another study, a transient induction of *Wnt10b* expression was seen in CMs located in the peri-infarct area. The researchers proposed that this response enhances angiogenesis and helps reduce fibrosis.^56^ Dissecting the functions of this signaling protein family in neonatal mouse regeneration models will be intriguing.

The Hh pathway plays an important role in vascular remodeling. When activated, it enhances the contribution of the epicardium to the development of coronary vessels and regulates the behavior of progenitor cells during the repair process.^23^ Since Hh signaling is involved in epicardium regulation in mammals during cardiac development, homeostasis, and regeneration, it has been proposed as a potential therapeutic target for coronary heart disease.^23^^,^^57^^,^^58^

The Hippo–YAP pathway has been proposed to regulate CM proliferation throughout mammalian life. Epicardial activation involves modulation of the Hippo pathway to enhance reparative outcomes.^59^ Indeed, several authors have reported dramatic effects on CM proliferation exerted by the derepression of YAP1 and its ability to bind target genes, even in the adult heart.^60–63^ Hippo pathway genes (*Yap*, *Taz*, *Tead1–Tead3, Lats1*, and *Lats2*) are also expressed in the proepicardium and epicardium during mouse heart development, and YAP1 and TAZ are required for vessel development in the epicardium.^64^  *Lats1* and *Lats2* deletion in mouse embryonic (E11.5) epicardium with the use of the *Wt1^CreERT2^* knock-in allele reduced fibroblast differentiation events.^65^ Single-cell RNA-sequencing assays suggested that *Lats1* and *Lats2* deletion preserved epicardial gene expression profiles and elevated the expression of YAP1 target genes, such as *Dhrs3* (a negative regulator of RA synthesis) and *Dpp4* (an ECM regulator). Whether the epicardial Hippo–YAP pathway is regulated by cardiac injury has not been investigated. As described above, deletion of *Yap* and *Taz* in the mouse epicardium induced profound pericardial inflammation, myocardial fibrosis, cardiomyopathy, and death after MI.^66^ This finding further supports that the epicardium mediates post-MI inflammatory responses.

Further, in post-MI mouse models, the activation of Notch signaling occurs in epicardial cells, CMs, and EPDCs.^67^^,^^68^ Inducible overexpression of the Notch1 intracellular domain in adult CMs following ischemic injury enhanced the percentage of CMs positive for the cell cycle active phase marker Ki67 but not for the proliferation marker phosphorylated histone 3; thus, the authors proposed a role for Notch signaling in promoting CM survival.^68^ Perhaps unsurprisingly, evidence from zebrafish and murine models highlights the necessity of tightly controlled Notch signaling activity to achieve beneficial effects in cardiac repair.

Neuregulin 1 (Nrg1), an extracellular factor implicated as an endothelial cell-derived mitogen for mammalian CMs,^69^^,^^70^ is a potent, inducible mitogen in zebrafish CMs. However, it is important to note that Nrg1 may stimulate only cell division in mononucleated and/or diploid CMs,^69^ potentially limiting the therapeutic Nrg1 efficacy in treating the damaged human heart. Existing evidence cannot exclude that the involvement of ERBB pathway ligands (other than Nrg1) could have ancillary or primary mitogenic activity in cardiac regeneration.

**Figure 2.**
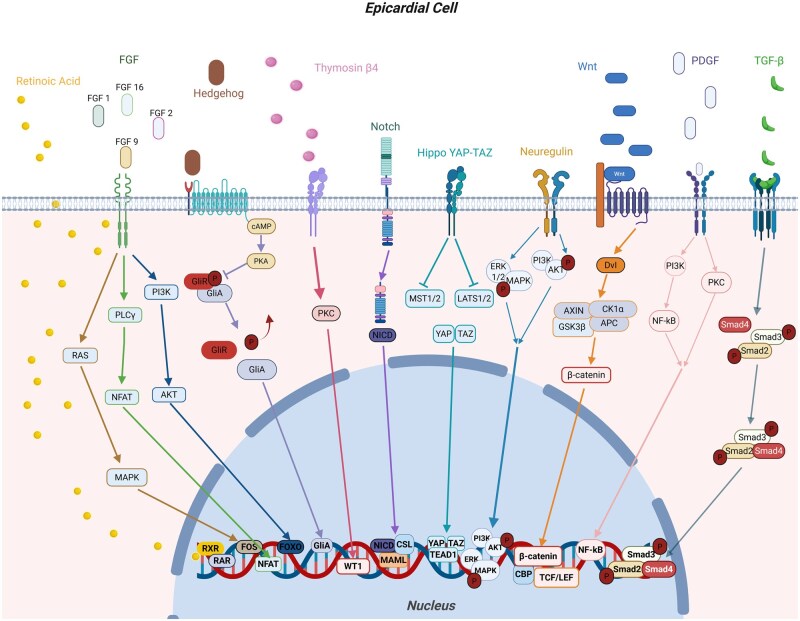
Intracellular signaling pathways in the epicardium regulate the molecular response to cardiac injury. After myocardial injury, the epicardium regulates the communication with the myocardium by secreting a broad array of signaling molecules, including Retinoic Acid (RA), Fibroblast Growth Factors (FGFs), Hedgehog, Thymosin β4, Notch, Hippo-Yes-associated protein/transcriptional coactivator with PDZ-binding motif (YAP/TAZ), Neuregulin, Wnt/β-catenin, Platelet-Derived Growth Factor (PDGF), and Transforming Growth Factor-β (TGF-β). These ligands bind to their specific receptors on the epicardial cell membrane and initiate intracellular cascades that ultimately converge in the nucleus to regulate gene expression. RA signaling regulates epicardial gene expression through nuclear retinoic acid receptors (RAR) and retinoid X receptors (RXR), controlling the transcription of key developmental genes and promoting the expression of FGFs, which in turn enhance cell survival, proliferation, and differentiation. Different fibroblast growth factor (FGF) family members activate the RAS/mitogen-activated protein kinase (MAPK), phospholipase C gamma (PLCγ)/nuclear factor of activated T cells (NFAT), and phosphoinositide 3-kinase (PI3K)/protein kinase B (AKT) pathways, which in turn regulate the transcription factors proto-oncogene (FOS), NFAT, forkhead box O (FOXO), respectively, thereby supporting myocardial growth and angiogenesis. Hedgehog signaling is activated through the Patched–Smoothened receptor complex. This results in the modulation of cyclic adenosine monophosphate (cAMP) levels, ultimately leading to the inhibition of protein kinase A (PKA)-mediated phosphorylation of GLI transcription factors. This allows full-length GLI activators to accumulate and translocate into the nucleus, where they contribute to coronary vasculature development and epicardial progenitor behavior. Thymosin β4 facilitates embryonic-like epicardial programs involved in neovascularization through activation of protein kinase C (PKC). This has been shown to initiate epicardial cell migration and activation of embryonic gene expression programs, including the re-expression of developmental transcription factors like Wilms’ tumor 1 (WT1). Notch signaling, triggered by ligand-receptor interactions, leads to the cleavage and nuclear translocation of the Notch intracellular domain (NICD), which combines with co-factors like mastermind-like protein (MAML) and CBF1/suppressor of hairless/Lag1 (CSL) to regulate genes involved in cell fate determination and inflammatory control. In parallel, the Hippo pathway controls the transcriptional co-activators YAP and TAZ, which are released upon inactivation of upstream kinases such as Mammalian STE20-like protein kinase 1 and 2 (MST1/2) and Large Tumor Suppressor Kinase 1 and 2 (LATS1/2). YAP/TAZ then translocate into the nucleus, together with TEA domain family member 1 (TEAD1), to promote genes involved in cell proliferation, regeneration, and vascular development. Neuregulin signaling, through epidermal growth factor (ERBB) receptors, activates the PI3K/AKT and extracellular signal-regulated kinase (ERK1/2 MAPK) pathways, supporting cardiomyocyte proliferation and survival. Wnt/β-catenin signaling, through the recruitment and activation of the intracellular scaffold protein Dishevelled (Dvl), stabilizes β-catenin by preventing its degradation via the glycogen synthase kinase 3 beta (GSK-3β), casein kinase 1 alpha (CK1α), adenomatous polyposis coli (APC), and AXIN complex. Stabilized β-catenin accumulates in the cytoplasm and translocates to the nucleus, where it binds T-cell factor/lymphoid enhancer-binding factor (TCF/LEF) transcription factors and coactivators like CREB-binding protein (CBP), modulating genes that drive epithelial-to-mesenchymal transition (EMT), fibroblast activation, and myocardial repair. PDGF signaling, through PI3K, PKC, and nuclear factor kappa B (NF-κB), stimulates EMT, vascular maturation, and matrix remodeling, whereas TGF-β plays a central role in inducing EMT via small mother against decapentaplegic 2/3 and 4 (Smad2/3/4)-mediated transcriptional regulation, facilitating the transformation of epicardial cells into mesenchymal derivatives. These epicardial responses are known to directly influence underlying cardiomyocytes and vascular cells in the myocardium through epicardial expression of target genes, leading to the secretion of growth factors, cytokines, and morphogens that diffuse into the myocardium and modulate cardiomyocyte proliferation, survival, angiogenesis, and inflammation. This dynamic interface regulates myocardial healing through a coordinated transcriptional and paracrine response. Created in https://BioRender.com.

Finally, other factors like Thymosin β_4_ (Tβ4),^71^ follistatin-related protein 1 (FSTL1),^72^ bone morphogenetic protein (BMP),^73^ and prokineticin receptor-1 (PKR1)^74^ are all reported to be involved in epicardium–myocardium interactions that facilitate cardiac repair (Figure 2).

## Therapeutic epicardial activation techniques

**Figure 3.**
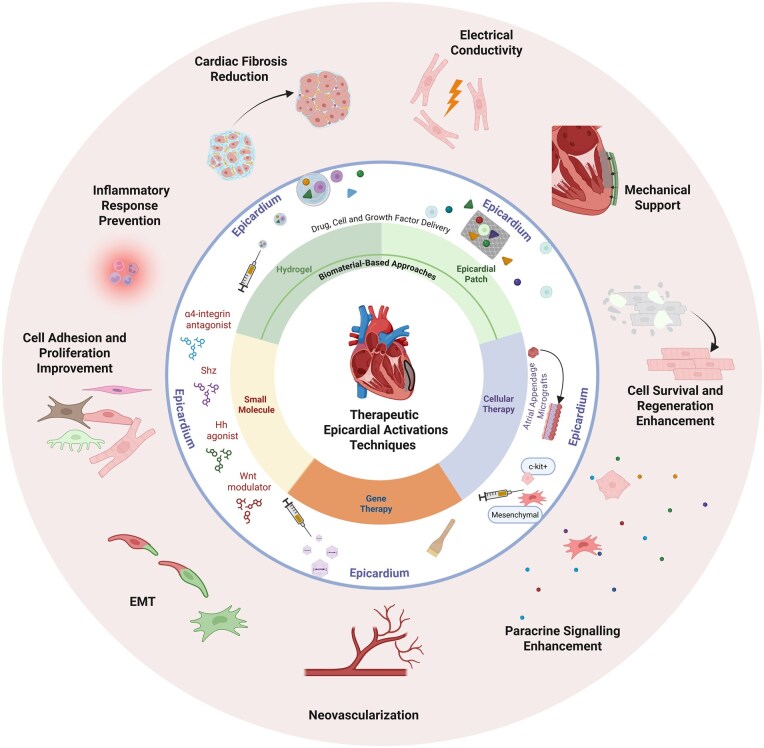
Comprehensive overview of therapeutic epicardial activation techniques to promote cardiac repair and regeneration. Small molecule therapies (bottom left) involve epicardial administration of compounds such as Wnt modulators, Hedgehog (Hh) agonists, sulfonyl-hydrazone (Shz) molecules, and α4-integrin antagonists. Gene therapy (bottom center) approaches deliver genetic material directly to the epicardium through injection or gene painting. On the right side, cellular therapy includes epicardial application of mesenchymal cells, c-kit+ cells, or implantation of atrial appendage micrografts. Biomaterial-based strategies, depicted in the upper half, encompass injectable hydrogels and epicardial patches, both enable localized delivery of therapeutic agents such as drugs, cells, and growth factors. Collectively, these interventions aim to induce epicardial epithelial-to-mesenchymal transition (EMT), enhance neovascularization, stimulate paracrine signaling, promote cell proliferation, and modulate inflammation. Improvement of electrical conductivity is a feature specific to biomaterial-based approaches, while the epicardial patches specifically provide mechanical support. Together, these strategies converge on reactivating developmental pathways in the epicardium to enhance survival, foster regeneration, and restore the functional performance of the injured heart. Epicardial activation, in turn, directly and indirectly influences the myocardial response to injury, contributing to its recruitment and participation in the reparative process. Created in https://BioRender.com.

Investigations into the epicardium’s role during heart development have provided insights into how reactivating embryonic programs in the adult epicardium could facilitate cardiac regeneration.^3^^,^^75^^,^^76^ Understanding these developmental processes offers potential therapeutic opportunities for enhancing the heart’s intrinsic repair mechanisms (Figure 3).

### Small molecule modulators

Small molecules targeting epicardial signaling pathways represent a versatile and non-invasive approach to reactivating the epicardium *in vivo*. Recent advancements in epicardial delivery systems pointed out the potential of small-molecule modulators in cardiac repair. These compounds can influence cellular pathways to promote heart tissue regeneration and improve function after injury.

For instance, the small molecule ICG-001 modulates Wnt/β-catenin signaling and enhances the expression of genes which promote cardiac regeneration in epicardial cells of chronically infarcted rat hearts.^77^ More recently, a small molecule screen revealed novel EMT inducers which may enhance epicardium-driven cardiac repair.^78^ Agonists of the Hh pathway stimulate epicardial cell activation and vascular remodeling, with positive effects on tissue perfusion and regeneration following cardiac injury.^23^ Further, a family of sulfonyl-hydrazone (Shz) small molecules has been identified for their ability to induce cardiac-specific gene expression in various stem and progenitor cells. When these Shz-enhanced cells were introduced into rat hearts near injury sites, they demonstrated improved cardiac function compared to control cells.^79^ Recently, small molecules have been utilized to efficiently obtain epicardial-like cells from human pluripotent stem cells^80^ and to lead to rat CM metabolic switching toward glycolysis/biosynthesis and CM de-differentiation before entering a proliferation stage.^81^

Manipulating epicardial ECM deposition may impact regeneration. For instance, α4-integrin signaling inhibition promoted epicardial EMT and affected epicardial lineage in a murine heart during development.^82^ The extracellular component hyaluronic acid and its receptor, hyaluronan-mediated motility receptor (Hmmr), are required for EMT and heart regeneration in zebrafish.^83^ Intriguingly, similar expression patterns were observed in the rat heart following infarction, suggesting their potential role in cardiac repair.^83^ Small molecules that modulate this process can enhance the epicardium’s reparative functions.

Ensuring precise delivery to the epicardium to avoid off-target effects and developing systems that provide sustained and controlled release of therapeutics are some of the most important challenges to harness the full potential of small molecule modulators in cardiac repair.

### Epicardial gene therapy

Gene therapy approaches aim to modulate epicardial signaling pathways to enhance cardiac repair directly. Delivery of genes via viral vectors, such as adeno-associated viruses (AAVs), has shown promise in activating the epicardium. Epicardial injection of the vector markedly enhances gene expression, which is limited to a few millimeters around the injection site, thus making a broader myocardial diffusion technically challenging.^84^ Another approach to successfully deliver vectors is epicardial gene painting. It consists of a combination of a gene vector, a poloxamer compound, and a protease (trypsin) that showed both safety and efficacy when applied to the epicardium.^85^

Overexpression of VEGF in the epicardium has been shown to promote neovascularization and improve myocardial perfusion. Interestingly, the EXACT trial (Epicardial Delivery of XC001 Gene Therapy for Refractory Angina Coronary Treatment) evaluated the safety and effectiveness of AAV-delivered VEGF-A gene via a minimally invasive epicardial approach in patients with refractory angina.^86^ The study showed that VEGF gene therapy with XC001 was safe and well-tolerated, with improvements in objective angina parameters.^86^ In addition, a small phase II clinical trial (NCT03370887) investigating the safety and tolerability of 30 epicardial injections of VEGF-A165 mRNA dissolved into citrate-buffered saline (AZD8601) in patients with reduced left ventricular function undergoing CABG appears to lay the groundwork for the clinical translation of this innovative therapeutic approach.^87^ A recent study has identified the critical role of the CCM2 gene in epicardial cells during heart development and repair. Investigations in zebrafish and mice have demonstrated that CCM2 is essential for proper cardiac development.^88^ In particular, CCM2 controls cytoskeletal and matrix gene expression, thus maintaining epicardial cell function and behaviors, and its deletion has been seen to affect EPDC differentiation and their migration within the myocardium, hampering cardiac regeneration. This result suggests that gene therapy targeting epicardial cells could enhance cardiac repair.^88^

### Epicardial cellular therapies

Epicardial injection is considered a reliable method for delivering therapeutic cells, resulting in higher cell retention within the myocardium compared to other delivery routes. This technique involves direct visualization and access to the epicardium, often performed during minimally invasive surgical procedures or in conjunction with coronary artery bypass grafting.^89^ Primary human adult EPDCs and their maintenance in an epithelial-like state were established a few years ago.^90^ The recent development of inducible proliferative adult human EPDCs (iEPDCs) provides a valuable model for studying epicardial cell characteristics.^91^ Further, epicardial-like cells (iECs) can also be derived from induced pluripotent stem cells (iPSCs) using a protocol based on temporal control of Wnt/β-catenin signaling.^92^ Studies are investigating the combined epicardial delivery of mesenchymal and c-kit+ cardiac stem cells to improve heart repair in patients suffering from ischemia-induced heart failure,^93^^,^^94^ and ongoing clinical trials are exploring the efficacy of epicardial delivery of cellular therapies.^95^ Recent research has focused on enhancing the regenerative capacity of EPDCs through genetic and pharmacological interventions^18^ as well as through paracrine signaling, mediated by epicardial extracellular vesicle (EV) release.^96^ In neonatal mouse hearts at postnatal day 7, which have lost their regenerative capacity after injury, the authors observed that cardiomyocytes re-entered the cell cycle in response to EVs treatment, thus effectively extending the regenerative window. This effect was mediated by the activation of the Akt, Hippo, and ERK signaling pathways. However, further studies are needed to assess the therapeutic potential of epicardial-derived EVs in the context of myocardial infarction (MI) in adult hearts. The authors suggested a role of some miRNAs identified in the EVs (miR-30, miR-21, and miR-100) with downstream targets involved in cardiomyocyte proliferation, like Tor and Notch.^96^

The feasibility and safety of autologous atrial appendage micrografts implanted onto the epicardium during coronary artery bypass grafting (CABG) surgery have been investigated. This approach seeks to promote myocardial regeneration through the direct application of micrografts to the epicardial surface, leveraging the high concentration of cardiac stem cells present in the right atrial appendages.^97^ The aim of this study was to evaluate the feasibility and safety of this strategy only; further investigation is needed to elucidate the mechanisms.

Although improvements were achieved, ensuring the long-term survival and functional integration of delivered cells into the host epicardium and developing strategies to control the host immune response to prevent rejection and enhance transplanted cell efficacy are the main challenges in this field.

### Biomaterial-based approaches

Biomaterials provide a platform for delivering epicardial activators in a controlled and sustained manner. Interestingly, the use of in situ biomaterial-based approaches can reduce off-target organ drug levels and maintain therapeutic effects over extended periods.

Injectable hydrogels that form cardiac patches in situ might offer a minimally invasive approach for cardiac repair. Recently, Zhu et al. showed in a murine MI model that, following pericardial injection, the hydrogel spread and formed a cardiac patch. The hydrogel-delivered MSC exosomes, taken up by epicardial cells, promoted an increase in WT-1+ and Ki67+ EPDCs in the epicardium, suggesting an epicardium-derived repair mechanism in the infarcted heart.^98^ However, this is the only study on hydrogel injection that focused on epicardial activation. Further investigations are required to confirm the effectiveness of epicardial hydrogel applications in cardiac repair and better elucidate the underlying mechanisms.

Another promising therapeutic strategy for cardiac repair consists of epicardial patches, which are designed to be applied directly to the heart’s surface, sometimes delivering cells, growth factors, or drugs to the damaged myocardium, thereby promoting tissue regeneration and functional recovery.^99^ Research has demonstrated that epicardial implantation of MSC-loaded patches promotes repair of the infarcted myocardium. These patches promote greater transplanted cell viability, accelerate paracrine signaling, activate the epicardium, and recruit endogenous c-kit+ cells, contributing to improved cardiac function.^100^ In particular, a higher number of WT1+ EPDCs was observed in the epicardium of the infarcted region, and some of these cells migrated into the myocardium after 4 weeks in the infarcted rat hearts transplanted with MSC-loaded epicardial patches compared to MSCs injected intramyocardially. Through lineage‐tracing approaches, these cells were found to differentiate into endothelial cells, vascular smooth muscle cells, and cardiomyocytes.^100^ Recruitment of c-kit^+^ cells was observed exclusively in hearts treated with the MSC-loaded patch, whereas no c-kit^+^ cells were detected following MSC injection alone. The authors excluded a mesenchymal origin of the c-kit^+^ cells.^100^ A more recent study reported that a lithium magnesium silicon (LMS)-containing cardiac patch activated the EMT process *in vitro*. After 4 weeks of LMS-patch *in vivo* transplantation, a high number of WT1+ cells co-expressed either CD31 or CD105, highlighting epicardial cell differentiation mainly into endothelial cells. RNA-seq analysis of heart samples showed epicardial (*Wt1*) and Notch pathway–related gene upregulation. These authors stated that LMS-patch-induced activation of the EMT program of epicardial cells occurs through the Notch pathway, inducing cardiac repair.^101^

Epicardial implantation of antioxidant polyurethane scaffolds combined with human amniotic epithelial stem cells has been shown to modulate the inflammatory environment post-myocardial infarction, improving cardiac function by influencing the immune response and directly favoring angiogenesis.^102^ Recently, a macrophage-mediated repair was reported using a biologically derived epicardial patch that enhanced the perfusion of the damaged area in a rat model of ischemic congestive heart failure, highlighting its potential for clinical application.^103^

Developments in ECM-based patches have shown promise for epicardial infarct repair. These bioactive patches, applied to the ischemic area, promote endogenous myocardial repair by providing structural support and biochemical cues that facilitate tissue regeneration.^104^ The mechanism proposed by the authors involves the release of paracrine factors (such as growth factors and matrix-bound vesicles) from the ECM into the subepicardium. These factors might mobilize resident repair cells and create a microenvironment favoring neoangiogenesis and cardiac repair.^104^ Innovative designs, such as intrinsically magnetic epicardial patches (MagPatch), have been developed for rapid vascular reconstruction and targeted drug delivery. These patches leverage magnetic properties to enhance their therapeutic efficacy and precision in cardiac repair applications.^105^

Very recent advancements have focused on improving the biomimicry, mechanical properties, and controlled release capabilities of bioartificial patch materials to further enhance their efficacy in epicardial activation and cardiac repair.^106^ In this study, the recruitment of c-kit+ cells and new vessel formation both inside the patch and in the infarcted area under the patch were consistently observed. In addition, a high number of GATA+ cells were found to be recruited in the patches, whose possible role in cardiac healing will need to be clarified.^106^

Overall, epicardial patches represent a versatile and evolving platform in regenerative cardiology, offering targeted delivery of therapeutic agents and structural support to the injured heart. Ongoing innovations continue to refine their design and functionality, bringing them closer to clinical application for patients suffering from myocardial infarction and other cardiac pathologies. Ensuring seamless integration of the patch with the native myocardium to facilitate effective electrical and mechanical coupling and developing cost-effective and reproducible manufacturing processes to produce patches suitable for widespread clinical use are the most critical forthcoming challenges.

### Therapeutic activation of aged epicardial cells

Aged epicardial cells have reduced regenerative capacity and altered cellular functions compared to their fetal or neonatal counterparts, often losing critical gene programs and becoming less secretory and mitogenic, which contributes to less effective heart repair in older adults.^107^^,^^108^ Age-related changes can also impact epicardial adipose tissue, leading to more pro-inflammatory and pro-fibrotic characteristics.^109^

Mitigation methods for aged epicardial cells, though not fully established, focus on promoting their youthful function by reactivating developmental programs,^9^ or enhancing repair processes through techniques like small molecule interventions, including GSK3α/β inhibitors^110^ and MAPK pathway inhibitors.^111^ Further approaches include metabolic switching promoting fatty acid-based metabolism through targeted drug therapies such as GLP-1 analogues^112^ or SGLT2 inhibitors for epicardial fat.^113^

## Challenges and future directions

Despite the growing body of evidence supporting the therapeutic potential of epicardial activation in cardiac repair, several challenges must be addressed before clinical translation becomes feasible.

Heterogeneity of epicardial responses is one of the challenges. The epicardium exhibits variable activation and functional contributions across individuals and species. This heterogeneity complicates the development of standardized and personalized therapeutic strategies. Interindividual variability, encompassing differences in genetic background, age, and disease state, influences epicardial activation. For instance, aged epicardial cells may exhibit reduced proliferative and regenerative capacity compared to those from younger individuals. Species-specific differences are also an issue, as studies in model organisms, such as zebrafish and neonatal mice, reveal robust epicardial contributions to regeneration. However, adult mammalian hearts display limited epicardial activation, necessitating strategies to overcome these intrinsic barriers. More precise identification of subpopulations of epicardial cells is also needed, as recent research indicates that the epicardium is composed of diverse subpopulations with distinct functional roles. Single-cell transcriptomics and lineage-tracing studies are uncovering these subpopulations, providing insights into their unique contributions to repair.

Incomplete differentiation of epicardial cells also remains a critical hurdle. Although EPDCs can differentiate into fibroblasts, smooth muscle cells, and potentially endothelial cells, their differentiation into functional CMs remains limited and controversial. Efforts to drive EPDCs toward a cardiomyocyte lineage have been met with limited success, even though advances in reprogramming technologies and signaling pathway manipulation, such as Wnt and Notch modulation, may enhance EPDC plasticity. Even in case EPDCs could be directed to differentiate into cardiac lineages, ensuring their functional integration into the myocardium remains challenging. Proper electrical coupling and contractile function are necessary for meaningful cardiac recovery.

Effective delivery of therapeutic agents to the epicardium remains a technical priority, particularly in minimally invasive clinical settings. Hydrogels, bioengineered patches, and nanoparticles have been developed to deliver therapeutic agents directly to the epicardium. These platforms provide localized and sustained delivery, reducing systemic exposure and enhancing efficacy. However, minimally invasive approaches are urgently needed. Innovations in catheter-based or laparoscopic delivery systems could make epicardium-targeted therapies more accessible to patients.

Finally, integrating epicardium-based therapies with existing treatments represents a critical need. For clinical adoption, epicardium-based therapies must complement or enhance current treatment modalities, such as pharmacological therapies, revascularization procedures, or mechanical assist devices.

Synergistic effects on myocardial repair might be obtained by combining epicardial activation with cardioprotective agents or angiogenic factors. Comprehensive preclinical investigation alongside well-planned clinical trials is crucial to validate the safety and therapeutic potential of epicardium-based treatments. Standardized outcome measures, such as scar size reduction and functional improvement, will facilitate comparisons across studies.

## Conclusions

In conclusion, epicardial activation represents a transformative approach to cardiac repair and regeneration, leveraging the inherent plasticity of the epicardium to support myocardial recovery. By understanding and manipulating epicardial biology, researchers aim to develop innovative therapies for heart disease, addressing an urgent unmet clinical need.

To realize the full potential of epicardium-based therapies, future research should focus on elucidating the molecular mechanisms underlying epicardial plasticity, developing smart biomaterials capable of responding to dynamic changes in the cardiac environment, an innovation that could revolutionize epicardial therapy delivery, and leveraging computational models of epicardial activation and cardiac repair. Along with comparative studies of epicardial function in regenerative species that may uncover novel targets and pathways for mammalian heart repair, this could provide valuable insights into the optimization of therapeutic strategies.

## Data Availability

No new data were generated or analyzed in support of this research.
